# Malaria treatment-seeking behaviour and recovery from malaria in a highland area of Kenya

**DOI:** 10.1186/1475-2875-7-245

**Published:** 2008-11-26

**Authors:** Peter O Sumba, S Lindsey Wong, Hemal K Kanzaria, Kelsey A Johnson, Chandy C John

**Affiliations:** 1 Kenya Medical Research Institute, P. O. Box 1578, Kisumu, Kenya; 2 Wake Forest University School of Medicine, Winston-Salem, North Carolina, USA; 3 University of California – San Francisco, San Francisco, California, USA; 4 Center for Global Pediatrics and Division of Pediatric Infectious Diseases, University of Minnesota Medical School, Minneapolis, Minnesota, USA

## Abstract

**Background:**

Malaria epidemics in highland areas of Kenya cause significant morbidity and mortality.

**Methods:**

To assess treatment-seeking behaviour for malaria in these areas, a questionnaire was administered to 117 randomly selected households in the highland area of Kipsamoite, Kenya. Self-reported episodes of malaria occurred in 100 adults and 66 children.

**Results:**

The most frequent initial sources of treatment for malaria in adults and children were medical facilities (66.0% and 66.7%) and local shops (19.0% and 30.3%). Adults and children who initially visited a medical facility for treatment were significantly more likely to recover and require no further treatment than those who initially went to a local shop (adults, 84.9% v. 36.8%, *P *< 0.0001, and children, 79.6% v. 40.0%, *P *= 0.002, respectively). Individuals who attended medical facilities recalled receiving anti-malarial medication significantly more frequently than those who visited shops (adults, 100% vs. 29.4%, and children, 100% v. 5.0%, respectively, both *P *< 0.0001).

**Conclusion:**

A significant proportion of this highland population chooses local shops for initial malaria treatment and receives inappropriate medication at these localshops, reslting in delay of effective treatment. Shopkeeper education has the potential to be a component of prevention or containment strategies for malaria epidemics in highland areas.

## Background

Malaria is a leading cause of death in children under the age of five years in sub-Saharan Africa [1]. The Roll-Back Malaria (RBM) initiative is working to improve prevention efforts in affected countries, through insecticide-treated nets (ITNs), indoor residual spraying (IRS) of pesticides, and intermittent preventive treatment (IPT) for pregnant women [2]. RBM also focuses on intervention efforts via effective anti-malarial regimens like artemesinin-based combination therapy (ACT), pre-empting epidemics in epidemic-prone areas, and improving home management of the disease. Rapid, effective treatment response with ACT is currently the most effective treatment option in sub-Saharan Africa, considering the current state of anti-malarial drug resistance with chloroquine and sulphadoxine-pyrimethamine [3], and artemether-lumefantrine is the now first-line treatment for malaria in Kenya [4]. Since many individuals in sub-Saharan Africa choose to treat malaria without visiting a medical facility, appropriate home management of malaria is vital to effective treatment of malaria.

A rapid treatment response is essential for effective home management. To this end, an understanding of treatment-seeking behaviour enables communities and the formal health care system to design interventions that cater to a specific population [5]. Unlike the inhabitants of areas of high endemicity, populations in highland East Africa are prone to large-scale malaria epidemics and generally lack protective immunity. All age groups are prone to severe malaria and death [6]. Despite the resulting high case fatality rates characteristic of epidemics, malaria treatment-seeking patterns in epidemic-prone areas of Africa are not well studied, particularly among adults. Limited information on treatment-seeking behaviour and anti-malarial use hinders the evaluation and implementation of effective malaria prevention and treatment programmes [7]. Some research efforts have pinpointed the need to train drug retailers about appropriate dosages and drug regimens, thus increasing adherence to national recommendations in the community [8]. Other studies have used survey instruments to assess knowledge, attitudes and practices (KAP) and to characterize actions taken by primary caregivers during malaria infection [5,9]. The present study utilizes the latter approach to study treatment-seeking behaviour among both adults and caretakers of children in a highland, epidemic region in Kenya.

One of the key strategic approaches outlined by the Kenyan National Malaria Strategy (KNMS) is the improvement of malaria epidemic preparedness and response [10]. With medical facilities, local shops and traditional healers available in Kipsamoite, Kenya, identifying where and how soon after individuals become symptomatic they seek treatment can help this community to identify current resource use. Financial and educational resources can then be better directed to improve the efficacy of interventional programmes.

## Methods

### Study site and population

Kipsamoite, Nandi District, is an epidemic-prone rural highland area with unstable malaria transmission. Altitude ranges from 1,910 m–2,154 m. The total population of the area is ~3,200 individuals. Study participants were recruited from all seven villages in the area. Subsistence farming is the principal occupation. More than 95% of the Kipsamoite population is of the Kalenjin Nandi tribe.

There are several small hospitals within 10 kilometers of the study site, though most people tend to go to the local medical facilities for health care. Kipsamoite health centre is centrally located and offers free malaria microscopy testing and medication. However, traditional herbalists and shops (*dukas*), offering over-the-counter medication, are frequently sought after alternatives to local health centres.

### Questionnaire development

A draft questionnaire of treatment-seeking behaviour for malaria and other diseases was created from open-ended (informal) key informant interviews. The initial questionnaire used was adapted from a Knowledge, Attitudes and Practices questionnaire used by the Centers for Disease Control in an area of urban malaria transmission [11]. It was then modified based on information provided by key informants. Key informants included field staff native to the study area, the chief and assistant chief of the area, teachers at the local schools, the microscopist at the medical health centre, textile merchants at the weekly market, and maize (corn) traders. These discussions were conducted to become familiarized with and gather contextual data of local health practices and travel habits. The questionnaire was then formulated and pilot tested on the field assistants, who were subsequently trained to administer it. The questionnaire was amended to make questions more culturally appropriate and use more familiar wording in the Kalenjin language. A pilot test on 12 randomly selected households was conducted to validate questionnaire reliability. The areas of questions asked in the questionnaire are outlined in the Results section. The study focused on self-reported malaria in adults and children in the households randomly selected for interview. Questions on malaria included questions in the areas of malaria knowledge ("Do you know about malaria") and whether the person had ever had malaria, using an area term ("cheptigonit") specifically used to refer to malaria. Individuals were asked to recall, for themselves or their household members, including children, whether they had ever had an attack of malaria, and if so, to describe, for the most recent episode details of how and where they were treated, what form of therapy they were given, and whether or not they recovered after receiving therapy. "Medical facility" was defined as a local facility staffed by personnel with medical or nursing training, which included the area health centre, as well as local dispensaries, clinics and hospitals. Symptoms included fever, headache, body ache, vomiting, weakness and diarrhea. Individuals were asked to grade each symptom according to a 3-point scale, with 1 being mild, 2 moderate and 3 severe symptoms. For dichotomous severity analysis, individuals were considered to have severe symptoms if they had at least two symptoms that they graded as severe. To assess the effects of distance from health centre, adults and children were classified into one of two groups depending on whether they lived less than 2 km from the local health centre or two or more km from the health centre. Economic status was measured by assigning each individual a wealth index. The wealth index was calculated as a composite number:

Wealth index = [(number of cows × 2) + number of sheep + number of goats + (number of acres of land owned × 2)]

The number of cows and the number of acres of land owned were weighted more heavily than other forms of property because cattle and land are the primary indicators of wealth in this community. Individuals were then ranked according to quartiles of wealth index. A number of individuals had the same wealth index number, resulting in quartiles with slightly greater or less than one quarter of the individuals.

### Participant recruitment and study design

Study participants were recruited from a continually maintained census database of Kipsamoite. The study was conducted in August-September, 2002, just after a malaria epidemic had occurred in the area [12]. A computer programme was used to randomly select 20% of households from within this area for participation in the study. Of the 120 households asked to participate, three refused, leaving 117 households for questionnaire administration. Questions were directed to the head of the household (male or female) about their treatment-seeking behaviour for themselves. Questions for any child of less than 12 years of age living in the household were directed to the child's primary caretaker, usually the child's mother. Respondents' accounts were case-based. Native Kalenjin speakers conducted each of the interviews. Literacy was not necessary for participation.

### Ethical review

This project was reviewed and approved by Case Western Reserve University/University Hospitals of Cleveland Institutional Review Board and the Ethics Committee of the Kenya Medical Research Institute, Nairobi, Kenya. The work was performed while the senior author (CCJ) was a faculty member at Case Western Reserve University. Informed consent was obtained from all participants.

### Data analysis

Frequencies and proportions were used for descriptive analysis. Since adults may behave differently when seeking treatment for themselves rather than their children, separate analysis was planned for adults vs. children as part of initial study design. Proportions were compared using Chi-square (χ^2^) tests of association to test for significant differences. Adjusted odd ratios, by multivariate logistic regression, were used to compare the likelihood of seeking malaria treatment at a medical facility and within 1 day of symptom onset according to symptom severity, distance from medical facility, and socio-economic factors. Interaction terms were included in logistic regression models. No significant interaction was seen in any regression model.

## Results

### Initial treatment source

Of the 120 households asked to participate, 117 agreed to administration of the questionnaire. Table 1 summarizes the time of episode and the initial treatment sought for self-described malaria. In the households surveyed, 100 adults and 66 children under the age of 12 reported episodes of malaria, where malaria was defined by a local term specific to the disease ("*cheptigonit*"). No household had more than two children with malaria reported, and only two households had two children with malaria – all other children with the only child in the household with reported malaria. Recent malaria episodes had occurred within six months of the interview more frequently in children than adults (90.9% v. 53.0%, *P *< 0.0001). Among symptomatic individuals, adults sought treatment within 1 day of symptom onset more often for themselves than for their children (68.0% v. 48.5%, *P *= 0.01). Medical facilities were the most common initial treatment source for both adults (66.0%) and children (66.7%). Local shops were the most popular alternative, providing initial treatment for 19.0% of adults and 30.3% of children. Frequencies of treatment seeking behaviour were similar regardless of the time between questionnaire administration and the malaria episode. For example, among adults who had malaria episodes, health care was initially sought care at a medical facility 50%, 64% and 67.9% of the time for one, two and six month periods from the malaria episode to questionnaire administration, while in children it was sought at a medical facility 66.7%, 60% and 67.2% for the same time periods. A small number of symptomatic adults and parents of symptomatic children used traditional healers or herbalists as initial sources of treatment. The majority of adults and children who did not recover after initial treatment went to a medical facility as their second line of treatment (22/28 adults, 78.6%, and 21/23 children, 91.3%). Thus, in the course of their illness, 88 of 100 adults (88.0%) and 65/66 children (98.5%) with self-diagnosed malaria went to a medical facility to seek treatment.

**Table 1:** Time of episode and treatment and initial treatment source of adults and children with self-described malaria.

	Adults (N = 100)	Children (N = 66)
	%	%
Malaria episode within 6 months of interview	53.0	90.9^a^
Treatment sought within 1 day	68.0	48.5^b^
Initial treatment source		
Health care facility	66.0	66.7
Shop	19.0	30.3
Traditional healer	4.0	1.5
Herbal medicine	5.0	0.0
Self treatment	4.0	0.0
No treatment	2.0	1.5
Ever treated at health facility	88.0	98.5

^a ^P < 0.0001, ^b ^P = 0.01, χ ^2 ^test.

### Treatment given for malaria

Table 2 summarizes recovery from self-described malaria according to initial health care provider and the treatment received. Sixty-eight of the 94 adults who received treatment and 51 of the 65 parents whose children received treatment recalled the treatment administered. At the time the study was conducted, sulphadoxine-pyrimethamine (SP) was the recommended first-line treatment in Kenya. Adults and children who initially visited a medical facility were much more likely to receive SP or another anti-malarial medication (quinine, amodiaquine or chloroquine) than those who visited local shops. Among individuals able to recall the medication given, all adults (51/51, 100%) and parents of symptomatic children (32/32, 100%) who went to a health facility named an anti-malarial medication, while only 5/17 adults (29.4%) and 1/19 parents of symptomatic children who went to a local shop (5.3%) recalled receiving an anti-malarial medication (*P *< 0.0001 for comparison of proportions for medical facilities vs. local shops, for both adults and children). Among the study participants who recalled receiving anti-malarial medications at the medical facility, chloroquine alone was received by only 4/51 adults (7.8%) and 3/32 children (9.4%); all others received medications recommended for malaria at the time of the study (SP, quinine or amodiaquine). Adults and children visiting local shops often received medicines without anti-malarial properties, such as acetaminophen, aspirin, anti-histamines, or antibiotics.

**Table 2:** Recovery from self-described malaria in adults and children, by initial health care provider and treatment given.

	Medical facility		Shop		Traditional healer/herbal medicine		No treatment	
Therapy	N	% Recovered^d^	N	% Recovered	N	% Recovered	N	% Recovered

Adults (N = 100)								
Anti-malarial^a^	51	86.3	5	80.0	0	0	0	0
No anti-malarial^b^	0	0	12	16.7	0	0	4	50.0
Not known^c^	15	80.0	2	50.0	9	77.8	2	0

All	66	84.9	19	36.8	9	77.8	6	33.3

Children (N = 66)								
Anti-malarial^a^	32	81.3	1	100	0	0	0	0
No anti-malarial^b^	0	0	18	38.9	0	0	1	0
Not known^c^	12	75.0	1	0.0	1	0	0	0

All	44	79.6	20	40.0	1	0	1	0

^a ^Sulphadoxine/pyrimethamine, quinine, amodiaquine, or chloroquine.

^b ^Paracetamol, aspirin, anti-histamine, antibiotics.

^c ^Respondent could not recall treatment or treatment content not known.

^d ^Percentage who reported recovery from symptoms with this treatment, with no requirement for further treatment.

### Recovery from malaria

Recovery from malaria was defined as self-reported recovery from symptoms with treatment, with no requirement for further treatment. Individuals who initially visited a medical facility were significantly more likely to recover from their illness than those who went to local shops for treatment (adults, 84.9% v. 36.8%, *P *< 0.0001; children 79.6% v. 40.0%, P = 0.002, respectively, Table 2). This was associated with the increased frequency of anti-malarial medication given at medical facilities as compared to local shops. The small numbers of adults and children who recalled receiving anti-malarial medication at a local shop had similar recovery rates (4/5, 80%, and 1/1, 100%, respectively, *P *> 0.99) to the adults and children who recalled receiving anti-malarial medication at a medical facility (44/51, 86.3% and 26/32, 81.3%, respectively, *P *= 0.54, Table 2). Interestingly the recovery rate for adults using herbal medicines or traditional healers (7/9 adults, 77.8%) was similar to that of adults going to medical facilities, although the number using these traditional treatment sources was relatively small.

Recovery rates of adults and children who attended a medical facility but could not recall what medication they received were essentially identical to those of the individuals who recalled receiving anti-malarial medication (12/15 adults, 80.0%, and 9/12 children, 75.0%, *P *> 0.99), suggesting that these individuals also received anti-malarial medication.

### Factors influencing treatment-seeking behaviours

Symptom severity, educational level and distance from medical facility did not affect the likelihood of seeking treatment at a medical centre (Table 3) or within one day of symptom onset (Table 4). Wealth index did not affect the likelihood of adults seeking treatment at a medical facility, but adults within the second and third wealth index quartiles were more likely to seek treatment for their children at a medical facility (Table 4). However, adults with greater wealth were less likely to seek treatment within one day of symptom onset, both for themselves and for their children (Table 4).

**Table 3:** Likelihood of initially seeking malaria treatment at a medical facility, according to symptom severity, distance and socioeconomic factors.

	Adults (N = 100)				Children (N = 63)^a^			
	
	N	OR^b^	95% CI	P	N	OR^b^	95% CI	P
Severe symptoms								
Absent	34	1			41	1		
Present	66	1.11	0.44, 2.80	NS	22	2.31	0.68, 7.74	NS
Education								
None	24	1			11	1		
Primary or higher	76	0.61	0.21, 1.74	NS	52	0.66	0.13, 3.31	NS
Distance from health centre^c^								
<2 km	58	1			29	1		
≥2 km	36	0.61	0.24, 1.54	NS	28	0.61	0.41, 4.58	NS
Wealth index								
1^st ^quartile	24	1			15	1		
2^nd ^quartile	20	1.41	0.37, 5.36	NS	12	5.71	0.92, 35.32	0.06
3^rd ^quartile	28	2.82	0.74, 10.77	NS	17	7.10	1.18, 42.88	0.03
4^th ^quartile	28	0.86	0.24, 2.93	NS	17	2.52	0.26, 10.02	NS

^a^Symptoms in children; other factors in caretakers. Information incomplete in 3 children.

^b^Adjusted odds ratio for seeking initial treatment at a medical facility vs. any other treatment source.

^c^Information on distance from health centre obtained for 94 adults and 57 children.

NS = not significant.

**Table 4:** Likelihood of seeking malaria treatment within 1 day of symptom onset, according to symptom severity, distance and socioeconomic factors.

	Adults (N = 100)				Children (N = 61)^a^			
	
	N	OR^b^	95% CI	P	N	OR^b^	95% CI	P
Severe symptoms								
Absent	47	1			38	1		
Present	53	0.52	0.20, 1.33	NS	23	1.54	0.47, 5.03	NS
Education								
None	24	1			9	1		
Primary or higher	76	1.43	0.46, 4.47	NS	52	2.99	0.59, 15.13	NS
Distance from health centre^c^								
<2 km	58	1			29	1		
≥2 km	36	0.84	0.31, 2.24	NS	28	1.47	0.46, 4.63	NS
Wealth index								
1^st ^quartile	24	1			15	1		
2^nd ^quartile	20	0.18	0.03, 1.01	0.05	12	0.42	0.07, 2.50	NS
3^rd ^quartile	28	0.12	0.02, 0.64	0.01	17	0.11	0.02, 0.68	0.02
4^th ^quartile	28	0.29	0.05, 1.65	NS	17	0.19	0.03, 1.10	0.06

^a^Symptoms in children; other factors in caretakers. Information incomplete in 5 children.

^b^Odds ratio for seeking treatment for malaria within 1 day of symptoms vs. waiting longer.

^c^Information on distance from health centre obtained for 94 adults and 57 children.

NS = not significant.

## Discussion

Early and appropriate treatment of malaria is essential to reducing the morbidity and mortality associated with this disease. Understanding treatment-seeking behaviour enables communities, national governments and international agencies to improve the efficacy of malaria interventions by implementing programmes tailored to a specific region. Unfortunately, treatment-seeking behaviours are poorly characterized in epidemic-prone areas of sub-Saharan Africa, particularly among adults, despite the severe morbidity and mortality associated with malaria epidemics. The present study in the epidemic-prone highland area of Kipsamoite, Kenya documents that although medical facilities are the primary source of initial care for malaria, local shops are a frequent alternative source of care. Individuals with symptoms consistent with malaria are rarely given anti-malarial medication at these shops, and this results in lower recovery rates.

This study was conducted prior to development of in-depth questions on malaria indicators and treatment in the Kenyan Ministry of Health Demographic and Health Surveys, which were initiated in the 2003 surveys. The questions in present study on treatment-seeking behaviour are more in-depth than the government survey, and the government survey focused on treatment of fever and convulsions in children, while the current study assessed treatment for a term specifically associated with malaria in the local community. Thus differences in anti-malarial treatment given may reflect in part the different outcomes assessed. For example, in the present study, 50% of children thought by their parents to have malaria received an anti-malarial drug for treament of malaria, whereas between 14.6% and 30.4% of children under 5 years of age with fever and convulsions received anti-malarial treatment in the government's 2003 survey [13]. Since in the present community, malaria is uncommon except during epidemics, it was felt that asking about malaria specifically, rather than fever and convulsions, which may be due to more diverse causes in this malaria hypoendemic area, was appropriate.

The data from the present study indicate that treatment-seeking behaviour in this area of highland Kenya differs from the behavioural patterns observed in regions of endemic malaria. In Kipsamoite, the most popular source of initial treatment was a health care facility for both adults and children. This differs from endemic regions, where medicines from local shops constitute the most popular first response to perceived malaria [14,15]. Although local shops were not the most popular treatment source in the Kenyan highland setting, they were the most frequently sought alternative to medical facilities, particularly for children. As a result, a significant portion of the population received initial treatment for perceived malaria from a local shop, although the proportion was lower than that previously observed in another low malaria transmission area of Kenya [16]. These findings indicate that, despite increased reliance on medical facilities in this highland area, local shops are an important treatment source and should be considered when designing treatment and intervention programmes.

The data also reveal that local shops generally provide medications without efficacy against malaria to individuals with symptoms of malaria, a phenomenon that also has been documented in other regions of the country. A study of the management of fevers in another region of seasonal transmission in Kenya found that a majority [77%] of visitors to shops received a medicine with no anti-malarial efficacy [16]. Surveys of treatment-seeking behaviours in three districts of Kenya with endemic malaria documented a similar excess of drugs without anti-malarial activity being distributed by local shops for the treatment of individuals with symptoms of malaria [14]. The tendency of shopkeepers to provide drugs without anti-malarial properties has implications for the effectiveness of malaria treatment. Recovery rates among adults and children who initially visited a medical facility for malaria treatment were significantly higher than for those who relied on local shops as their initial source of treatment, and the data suggest that this was a result of the distribution of anti-malarial drugs at medical facilities but not at local shops. Most individuals who did not recover from their initial treatment used medical facilities as a second-line alternative so the vast majority of the population eventually sought treatment from a medical facility, where they received anti-malarial drugs. However, this resulted in delayed effective treatment for the individuals who first sought care at the local shops. With a significant proportion of the highland population relying on shops for their initial treatment, there is potential to improve malaria care by educating local shopkeepers on the symptoms and appropriate treatment of malaria. Such educational interventions have been shown to change malaria treatment-seeking behaviour in other malaria endemic areas [17,18]. In addition, ensuring that shopkeepers understand the proper drug regimen for treating an episode of malaria may help protect against the development of drug resistance by limiting over-use and under-dosing [14]. Finally, education of shopkeepers has the potential to assist significantly in prevention or containment of epidemics. Rapid, appropriate treatment for malaria is a mainstay of epidemic prevention. Since shops constitute an important source of malaria treatment in this area, shopkeeper dispensing of appropriate anti-malarials to individuals with malaria during an epidemic period might contribute significantly to reduction of malaria spread during the early stages of an epidemic.

Within this Kenyan highland population, the decision to seek treatment at a medical facility or to seek treatment within the first 24 hours of symptom onset was independent of perceived symptom severity, education level and distance from the health centre. These findings contrast with those of other studies that document that accessibility of health facilities and disease severity affect treatment-seeking patterns [15,19]. The relatively small area of study and the established nature and quality of the medical facilities in this area may have obscured the effects of distance on health-seeking behaviour. Similarly, the imprecise nature of self-reported severity of symptoms and the degree to which this might differ according to individual perception might affect the accuracy of the present study findings regarding severity of symptoms. Nonetheless, additional research in this area is merited, since the overemphasis of economic considerations can divert attention from the cultural factors and structural constraints influencing treatment-seeking decisions [20].

The present study was conducted at a time when SP was the recommended first-line drug for malaria in Kenya. With SP resistance, the ACT medication artemether-lumefantrine is now the recommended first-line drug for malaria treatment in Kenya. It is recommended that artemether-lumefantrine be dispensed only to individuals with blood-smear documented malaria, to avoid overtreatment of other causes of fever with ACT, and thus decrease the likelihood that drug resistance to ACT will develop. This approach raises further issues for potential interventions, particularly in highland areas. If shopkeepers are an important source of health care for individuals in these areas with symptoms of malaria, should they be educated to recommend ACT when an individual comes to their shop with symptoms of malaria? Will this be a successful intervention when ACT is considerably more expensive than other anti-malarial medications such as SP, amodiaquine and quinine? If the intervention is successful, will this result in an increase in drug-resistant *P. falciparum *in this area? It could be argued that this phenomenon is much less likely to occur in highland areas than in malaria holoendemic areas because transmission levels and frequency of asymptomatic parasitaemia are very low in highland areas [12]. Nonetheless, the necessary use of ACT in this and other areas of Kenya further complicates the issue of whether shopkeeper education will improve efforts to rapidly and effectively treat malaria in highland areas.

Like all questionnaire-based studies, this study is limited by the self-reported data, which are susceptible to recall and reporting biases. Attempts were made to minimize this potential source of bias by pilot testing the questionnaire, training field assistants in its administration, and amending it to make the wording familiar and culturally appropriate. As in other questionnaire studies of malaria, the occurrence of malaria was assessed by a local term that translates as "malaria", but the symptoms of malaria (fever, headache, body ache, vomiting, weakness and diarrhea) might well have been due to other diseases in some cases. However, the high frequency of recalled response to medications with efficacy against malaria and the low frequency of recalled response to medications with no anti-malarial efficacy suggest that malaria was an accurate diagnosis in a majority of the cases. In this study, questions were asked of the household head or, for children, to the caretaker of the child. Since household heads may have greater access to care than other household members, this could have created a bias in the data obtained. Ideally, all household members should have been asked about malaria symptoms, but resource and time limitations precluded this preferred approach. Nonetheless, even if the data are not completely representative, the pattern of failure with treatment provided by shops is a significant and important finding, and likely reflects the experience of most in the community. Finally, malaria is infrequent in these areas in non-outbreak periods, so recall of exact malaria treatment could be difficult, particularly for individuals whose episode was >6 months prior to the questionnaire. Questions are ideally asked within weeks of the occurrence of the disease, but in this area this was not possible, as it would be very difficult to get adequate numbers of individuals with disease within 1 month of the questionnaire. This is demonstrated by the finding that although the questionnaire was administered shortly after an epidemic in the area, the majority of adults had still not had an episode of malaria during the prior 2-month epidemic period. Ideally, the survey should have been done in a much larger population to allow for a sufficient sample size to evaluate treatment seeking-behaviour within the prior 14 days. The limited resources available for conducting the present study precluded this more optimal testing. It seems likely that the data was accurate despite the variability in time from malaria episode to questionnaire administration for two reasons. First, the treatment options for malaria are limited, both in terms of places to seek care and types of treatment, so recalling what was done from this limited number of choices would not be particularly difficult. Second, the data on treatment-seeking in both children and adults was highly consistent over all time periods (2 weeks, 1 month, 6 months, >6 months), supporting the idea that recall was accurate even over longer time periods.

## Conclusion

The present study in an epidemic-prone area of highland Kenya indicates that individuals in this area frequently sought treatment for malaria from local shopkeepers, and that treatment provided by these shopkeepers rarely had efficacy against malaria, leading to lack of recovery and delayed effective treatment for malaria. There may be an opportunity to improve the management and response to malaria epidemics by educating shopkeepers on the importance of prompt treatment of individuals with malaria symptoms with appropriate anti-malarial medication.

## Competing interests

The authors declare that they have no competing interests.

## Authors' contributions

POS was responsible for data analysis and interpretation and for the initial manuscript draft. LW and HK were involved in study design, data collection and revision of the manuscript. KJ was involved in data analysis, interpretation and critical revision of the manuscript. CCJ was responsible for study design and conception and involved in data analysis, interpretation and critical revision of the paper for intellectual content.
